# Higher Post‐Operative L‐Thyroxine Dosage Needed After Right Compared to Left‐Lobe Hemithyroidectomy

**DOI:** 10.1002/edm2.70345

**Published:** 2026-09-22

**Authors:** Carola Deischinger, Michael Krebs, Thomas Scherer, Christian Scheuba, Alexandra Kautzky‐Willer, Lana Kosi‐Trebotic

**Affiliations:** ^1^ Gender Medicine Unit, Clinical Division of Endocrinology and Metabolism, Department of Internal Medicine III Medical University of Vienna Vienna Austria; ^2^ Division of Endocrine Surgery, Department of General Surgery Medical University of Vienna Vienna Austria

**Keywords:** hemithyroidectomy, levothyroxine, lobectomy, postoperative levothyroxine dosage, thyroid

## Abstract

**Purpose:**

Usually, the right lobe of the thyroid gland is larger than the left lobe. Post hemithyroidectomy, the remaining lobe grows by up to 30% as compensation. Thyroxine replacement is still necessary for 22%–60% of all patients. We aimed at investigating whether a right‐lobe hemithyroidectomy requires higher postoperative levothyroxine dosages.

**Patients and Methods:**

426 adult patients who received a hemithyroidectomy and a follow‐up visit (median 9 months post‐surgery) at the Medical University of Vienna between 1994 and 2018 were identified and investigated in this retrospective study with a weight and pre‐surgery TSH‐adjusted model.

**Results:**

Patients required a higher mean levothyroxine dosage after right‐lobe hemithyroidectomy (mean = 68.1 μg/day, *p* = 0.018) compared to the left lobe (mean = 60.2 μg/day).

**Conclusion:**

Patients require roughly 13% more levothyroxine substitution after hemithyroidectomy of the right lobe compared to the left lobe, which should be considered in post‐op care.

## Graphical abstract

Patients required a 13.2% higher mean levothyroxine dosage after right‐lobe hemithyroidectomy (mean = 68.1 μg/day, *p* = 0.018) compared to a left‐lobe hemithyroidectomy (mean = 60.2 μg/day).

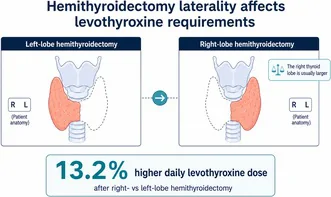

## 1 Introduction

Thyroid hormones, thyroxine (T4) and triiodothyronine (T3), are essential to human metabolism, influence the basal metabolic rate, bone growth, neural maturation, the body's sensitivity to catecholamines, cell development and differentiation as well as protein, fat and carbohydrate metabolism. The maintenance of adequate thyroid hormone levels is crucial for the patient's energy levels and quality of life after thyroid surgery [1, 2]. The thyroid consists of a right and a left lobe, which are connected via the isthmus. In most patients, the right lobe is significantly larger than the left lobe [3]. In a 2009 study by Ying and Yung, the right lobe was 6.8 mL as opposed to the left lobe with a volume of 5.7 mL [4]. Although the remaining lobe adapts and continues to grow by up to 30% from its original size to compensate for the loss due to lobectomy [5], thyroid hormone supplementation is required in approximately 22%–60% of all patients after hemithyroidectomy [6, 7, 8, 9, 10, 11, 12]. In order to predict the necessity of thyroid hormone replacement therapy and optimize post‐hemithyroidectomy levothyroxine dosing, previous studies incorporated pre‐surgery thyrotropin (TSH) levels, sex, age, body surface area (BSA) and body mass index (BMI), but not whether resection of the right or the left thyroid lobe is a factor [13, 14]. Therefore, our study investigates a possible difference in postoperative levothyroxine doses depending on the side of the resected lobe.

## 2 Materials and Methods

### 2.1 Study Participants and Design

**FIGURE 1:**
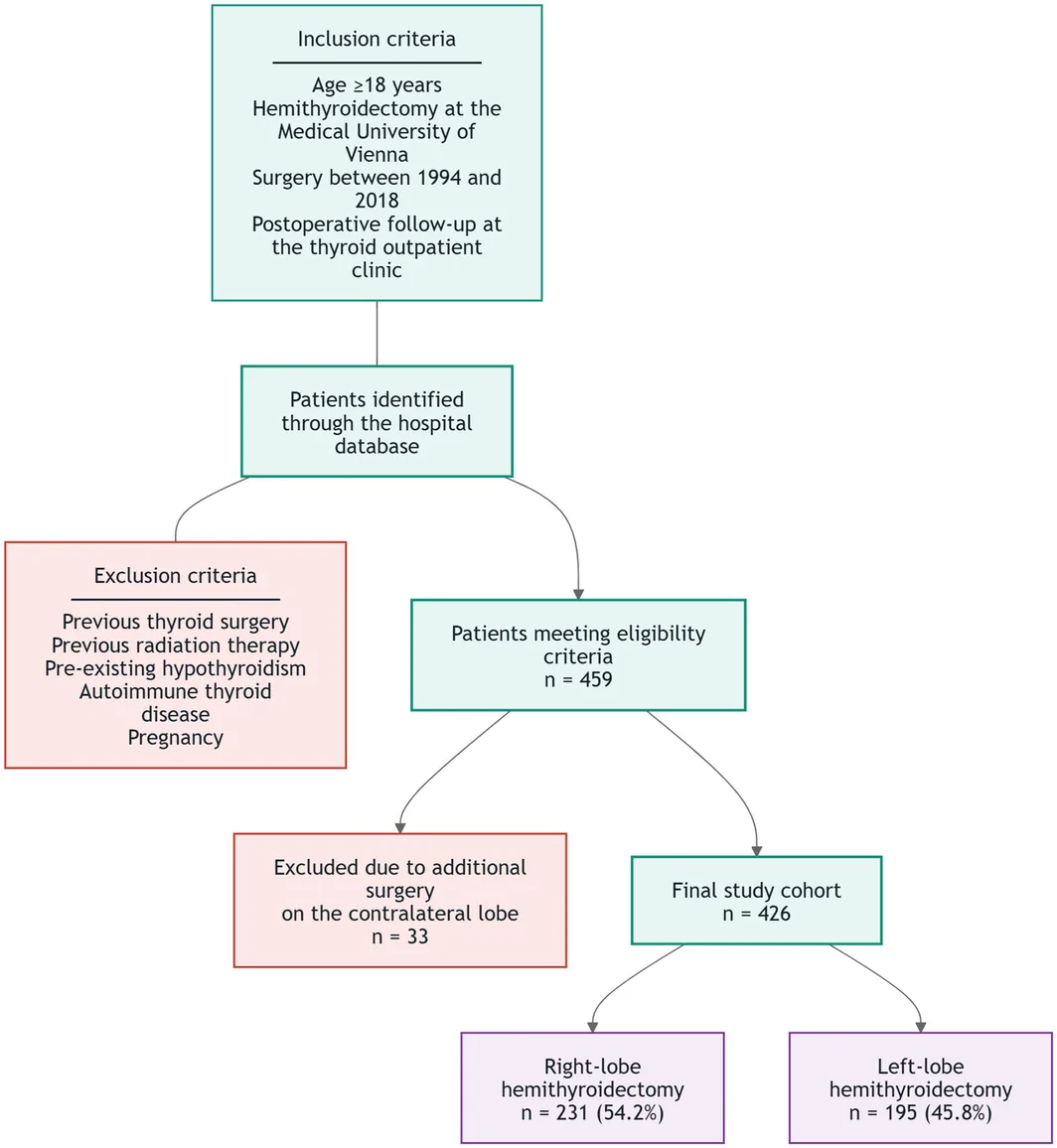
Patient selection flowchart. Of 459 patients meeting the inclusion and exclusion criteria, 33 were manually excluded because of an additional surgery on the contralateral lobe. The final study cohort comprised 426 patients, including 54.2% (231 patients) who underwent a right‐lobe hemithyroidectomy and 45.8% (195 patients) after left‐lobe hemithyroidectomy.

In a retrospective cohort study, 459 patients over the age of 18 years who underwent a hemithyroidectomy at the Department of General Surgery, Division of Endocrine Surgery and a follow‐up (9 months post‐surgery) visit at the thyroid outpatient clinic of the Department of Endocrinology and Metabolism between 1994 and 2018 were investigated. The work has been reported in line with the STROCSS criteria [15]. The patients were identified via data extraction from a university hospital wide Research, Documentation and Analysis (RDA) database. In a second step, further information necessary for the analysis (ultrasound data on pre‐surgery thyroid volume, pre‐surgery TSH, side and date of hemithyroidectomy, surgery protocol, underlying thyroid disease, type and dosage of treatment, age, weight, height, thyrotropin (TSH), free T4 (fT4), free T3 (fT3)) were extracted from the internal electronic health record system. Patients who had either received radiation therapy, had undergone previous thyroid surgery, surgery on the contralateral lobe, had pre‐existing hypothyroidism, an autoimmune disease of the thyroid or were pregnant were excluded from the analysis. Thirty‐three patients were excluded due to additional surgery on the contralateral lobe resulting in a total of 426 patients (see Figure 1). Pre‐surgery ultrasound thyroid volume was calculated with the volumetric ellipsoid method (height × width × depth × correction factor 0.524) [16].

### 2.2 Statistical Analysis

Patient baseline characteristics were described as percentages and frequencies for categorical variables or mean and standard deviation for normally distributed continuous variables. Differences of characteristics between the groups were analysed with unpaired, two‐sided *T*‐tests or Mann–Whitney *U* tests for continuous variables/distributions and a Chi‐squared test for categorical variables. As the weight had not been recorded for 21 of the 426 patients, regression imputation was used to estimate the missing variables. Kolmogorov–Smirnov and Shapiro–Wilks tests were used to assess normal distribution. A Mann–Whitney *U* test (unadjusted) and a multiple linear regression with HC3 heteroskedasticity‐robust standard errors adjusting for the potential confounding factors weight and pre‐surgery TSH were conducted to compare the levothyroxine dosages/distributions. The analyses were repeated with subgroups differentiating between hemithyroidectomy due to goitre or adenomas. Spearman's correlation was used for correlation analysis. Parameters with *p* < 0.05 were entered into a multiple regression analysis for detecting independent associations with levothyroxine dosage. Multicollinearity was tested in all linear regression models. All entered variables were checked for normality with normal probability plots and homoscedasticity using scatter dot plots. The collected data was analysed and illustrated in SPSS 25.0 (SPSS Inc., Chicago, USA) by using graphs, tables and boxplots.

## 3 Results

### 3.1 Patient Characteristics

With 75.4% females, the patients were predominantly women. 91.3% of all patients who had undergone surgery and went for a follow up to the thyroid clinic developed hypothyroidism after surgery. With 93% compared to 89.2%, the rate of hypothyroidism was slightly, albeit not significantly, higher in patients after right‐lobe hemithyroidectomy. Baseline characteristics such as age, height and weight were similar in both groups (see Table 1). The volume of the resected lobe was 30% greater in the right‐lobe than left‐lobe hemithyroidectomy group (*p* = 0.041); however, the volume of the resected lobe was only available in 112 patients. The post‐surgery blood sample taken at the thyroid clinic showed higher TSH levels in patients after right‐lobe hemithyroidectomy (1.70 IQR 1.01–2.76 μIU/mL) vs. left‐lobe hemithyroidectomy (1.40 IQR 0.91–2.26 μIU/mL; *p* = 0.011). After surgery, the median interval until the visit in the thyroid clinic after right‐lobe hemithyroidectomy was 9.03 months (IQR 4.06–12.39), 8.67 months after left‐lobe hemithyroidectomy (IQR 3.04–13.5), with no group difference (*p* = 0.943).

**TABLE 1:** Patient characteristics at baseline and post hemithyroidectomy of the left compared to the right lobe.

	All (*n* = 426)		Hemithyroidectomy right lobe (*n* = 231)		Hemithyroidectomy left lobe (*n* = 195)		*p*
	Mean/Median	SD/IQR	Mean/Median	SD/IQR	Mean/Median	SD/IQR	
Baseline							
Sex (% of females)	75.4%		73.2%		77.9%		0.152
Age (years)	52.9	15.3	53.5	15.3	52.3	15.3	0.440
Height (cm)	167	9	167	9	167	9	0.622
Weight (kg)	76.0	17.2	76.1	16.2	75.8	18.3	0.834
Pre‐surgery TSH (μIU/mL)	1.42	0.89–2.13	1.47	0.89–2.30	1.37	0.87–1.90	0.253
Volume of resected lobe (mL, 112 patients)			10.1	6.9–18.6	7.6	5.2–15.0	**0.041**
Post‐surgery							
Levothyroxine dosage (μg/day)	65.0	36.5	68.7	37.6	60.6	34.8	**0.033**
TSH (μIU/mL)	1.54	0.99–2.53	1.70	1.01–2.76	1.40	0.91–2.26	0.011
fT4 (ng/dL)	1.39	0.25	1.39	0.27	1.40	0.24	0.714
fT3 (pg/mL)	2.90	0.49	2.87	0.46	2.94	0.53	0.310

*Note:* Continuous variables were summarized by mean ± standard deviation (SD) or median and IQR (interquartile range) and categorical variables by percentages. To assess differences between the two groups hemithyroidectomy of left and right lobe, Chi‐squared tests, unpaired, two‐sided *T*‐tests and Mann–Whitney *U* tests were performed. Bold *p*‐values indicate a significance level *p* < 0.05.

Abbreviations: fT3, free triiodothyronine; fT4, free thyroxine; TSH, thyroid stimulating hormone.

### 3.2 Post‐Op Levothyroxine Dosage Needs

**FIGURE 2:**
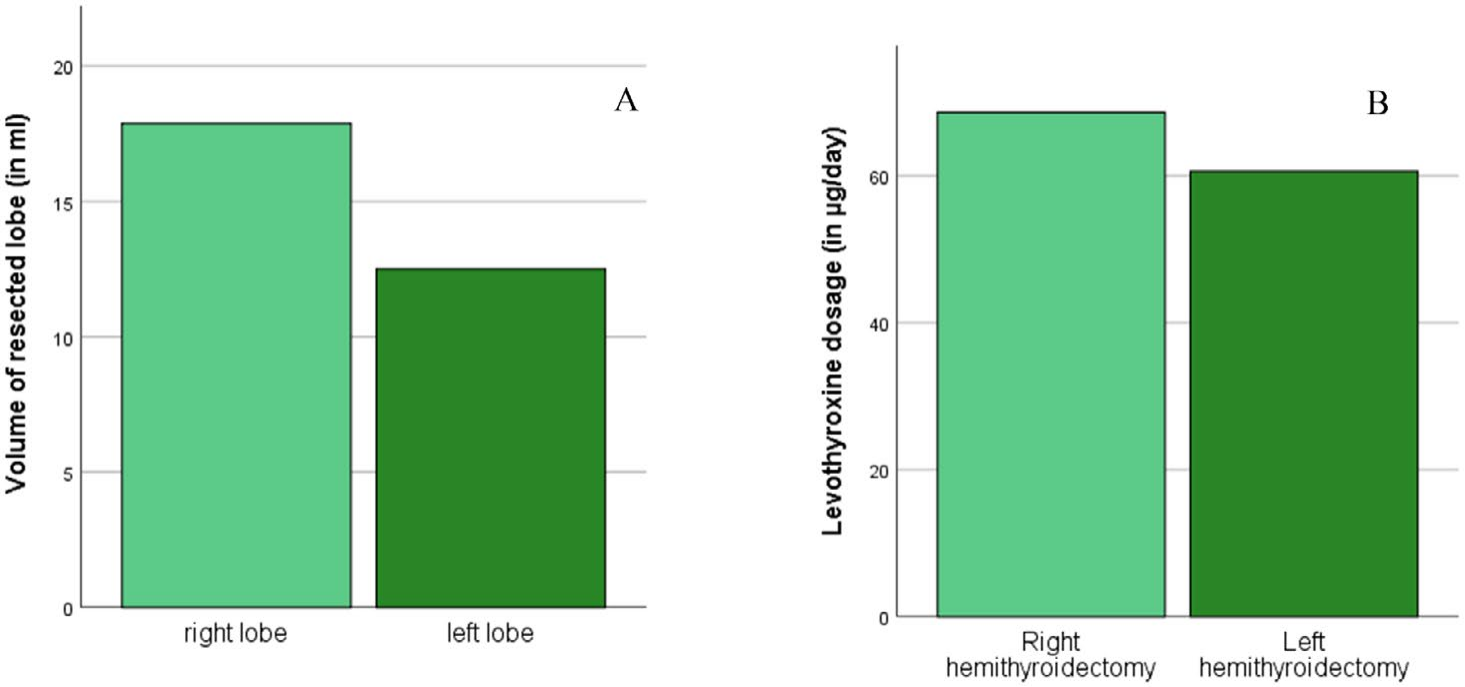
Volume of resected lobe in mL (A) compared to thyroxine dosage after right or left hemithyroidectomy in μg/day (B).

Right‐sided hemithyroidectomy was associated with a 13.2% higher adjusted levothyroxine dose compared with left‐sided surgery (+7.92 μg/day; 95% CI 1.38–14.45; *p* = 0.018) (see also Figure 2A,B). The levothyroxine dosage of patients after right‐lobe hemithyroidectomy was 68.0 ± SD 37.6 μg/day, which is statistically higher (*p* = 0.033) than in patients after left‐lobe hemithyroidectomy (60.6 ± SD 34.8 μg/day). In a weight and pre‐surgery TSH adjusted regression model, the mean levothyroxine dosage of patients after right‐lobe hemithyroidectomy was 68.1 μg/day, which is statistically higher (*p* = 0.018) than in patients after left‐lobe hemithyroidectomy (60.2 μg/day).

The levothyroxine dosage normalized to body weight, the median dose was 0.840 μg/kg/day (IQR 0.645–1.155) after right‐lobe hemithyroidectomy and 0.773 μg/kg/day (IQR 0.588–1.039) after left‐lobe hemithyroidectomy (*p* = 0.025).

In the subgroup of patients with goitre, the adjusted dose was 16.0% higher after right‐sided surgery (+9.37 μg/day; 95% CI 0.08–18.66; *p* = 0.048). In the group of nodules and autonomous adenomas, the adjusted difference was 9.7% (+6.0 μg/day; 95% CI −2.86 to 14.92; *p* = 0.182).

An exploratory Spearman's correlation analysis showed that levothyroxine dose was associated with the side of the hemithyroidectomy, preoperative TSH and weight, whereas sex and age did not impact the results (see Table 2). In the adjusted regression model, surgical side (7.69 μg/day, 95% CI 1.17–14.21, *p* = 0.021), body weight (0.38 μg/day, 95% CI 0.16–0.58, *p* < 0.001), and preoperative TSH (7.65 μg/day, 95% CI 3.27–12.02, *p* < 0.001) remained significantly associated with levothyroxine dose.

**TABLE 2:** Spearman's correlation analysis of post‐surgery levothyroxine dosage and potential covariates.

Spearman's correlation analysis	*R*_S_	*p*
Side of surgery (left or right lobe)	**−0.103**	**0.033**
Pre‐surgery TSH	**0.229**	**< 0.001**
Weight	**0.177**	**< 0.001**
Height	0.037	0.461
Age	0.075	0.123
Sex	−0.057	0.243

*Note:* Bold *p*‐values indicate a significance level *p* < 0.05.

Abbreviation: TSH, thyroid stimulating hormone.

## 4 Discussion

Patients post right‐side hemithyroidectomy were prescribed a 13% higher mean levothyroxine dosage than patients after left‐lobe hemithyroidectomy. The thyroid tissue loss was 30% greater in the right‐lobe than left‐lobe hemithyroidectomy group. Side of hemithyroidectomy, weight and pre‐surgery TSH levels were significant predictors of the recorded post‐surgery levothyroxine doses in this study.

The higher levothyroxine need after hemithyroidectomy of the right lobe might be explained by the differing sizes of the lobes. The right thyroid lobe is usually larger than the left lobe [4], which we were able to corroborate with pre‐surgery US data on thyroid lobe volumes. Lang et al. connected the size of the resected thyroid lobe to post‐surgery hypothyroidism risk in a previous study. Their study determined that patients with a BSA‐adjusted volume smaller than 3.2 mL of the contralateral lobe were associated with a three times greater risk to develop hypothyroidism [8]. In contrast to the study at hand, levothyroxine need and dosage were not investigated in this study [8]. Our analysis showed that patients post hemithyroidectomy of the right side might need more thyroxine than patients with left lobe resection in a weight and pre‐surgery TSH level‐adjusted model. Incidence of post‐surgery hypothyroidism was slightly higher amongst patients after right‐side hemithyroidectomy, although this was not statistically significant. Sex of the patient did not affect the results in respect to thyroxine doses although 75.4% of the patients were female. The high rate of female patients was to be expected due to the susceptibility of women to thyroid diseases [17].

A limitation of this study is that we initially identified 459 patients, who underwent a hemithyroidectomy and a follow‐up at our thyroid outpatient clinic; thus, most likely, not representing all hemithyroidectomy patients of the surgery department. This might explain the high observed prevalence of hypothyroidism in the cohort. A possible confounding factor is that the underlying conditions precipitating the surgery, such as goitre or nodules, might influence the results. We included a sub‐analysis differentiating between patients with goitre or nodules; however, many patients might have had both nodules and goitre, making a distinction difficult. Furthermore, the number and exact timing of the levothyroxine dosage adjustments preceding the follow‐up visit were not systematically recorded in all patients; the titration process itself could not be analysed. We rigorously excluded patients with any surgery on the contralateral lobe to obtain reliable results. Further strengths of our analysis include the large number of patients and the long observation period.

Previous studies used weight, pre‐surgery TSH levels, sex, age, BSA and BMI to build models to improve or systematize the prediction of the necessity of post‐hemithyroidectomy levothyroxine therapy [13, 14]. Jin et al. argued that weight is the best predictor of post‐surgery levothyroxine therapy needs [13]. Although weight was significantly related to post‐operative levothyroxine dosage needs in this analysis as well, our regression model identified side of hemithyroidectomy, pre‐surgery TSH and weight as significant predictors.

## 5 Conclusion

As hypothesized, patients need more levothyroxine substitution after hemithyroidectomy of the right lobe compared to the left lobe. If available, a pre‐surgery ultrasound to determine the size of the remaining lobe might be especially sensible if the right lobe is planned to be resected. Further randomized controlled studies are necessary to confirm our results in a prospective setting and improve current prediction models of post‐surgery thyroxine dosage needs. Consequently, the side of the hemithyroidectomy might have an impact on post‐op care as patients after right‐side hemithyroidectomy might require more thyroxine substitution than those after left‐side hemithyroidectomy.

## Author Contributions

**Carola Deischinger:** conceptualization, methodology, software, data curation, investigation, validation, formal analysis, funding acquisition, visualization, project administration, writing – original draft. **Michael Krebs:** validation, supervision, writing – review and editing. **Thomas Scherer:** validation, supervision, writing – review and editing. **Christian Scheuba:** writing – review and editing, resources, supervision. **Alexandra Kautzky‐Willer:** supervision, resources, writing – review and editing. **Lana Kosi‐Trebotic:** conceptualization, supervision, writing – review and editing.

## Funding

The authors have nothing to report.

## Conflicts of Interest

The authors declare no conflicts of interest.

## Data Availability

The data that support the findings of this study are available on request from the corresponding author. The data are not publicly available due to privacy or ethical restrictions.
